# Incidence and cost of anal, penile, vaginal and vulvar cancer in Denmark

**DOI:** 10.1186/1471-2458-12-1082

**Published:** 2012-12-17

**Authors:** Jens Olsen, Tine Rikke Jørgensen, Kristian Kofoed, Helle Kiellberg Larsen

**Affiliations:** 1 Centre for Applied Health Services Research and Technology Assessment (CAST), University of Southern Denmark, J. B. Winslows Vej 9B, 5000, Odense C, Denmark; 2 Sanofi Pasteur MSD ApS, 2800 Kgs., Lyngby, Denmark; 3 Department of Dermato-Venereology, Copenhagen University Hospital, Bispebjerg, Denmark

**Keywords:** Anogenital, Cancer, Cost, Incidence, HPV

## Abstract

**Background:**

Besides being a causative agent for genital warts and cervical cancer, human papillomavirus (HPV) contributes to 40-85% of cases of anal, penile, vaginal and vulvar cancer and precancerous lesions. HPV types 16 & 18 in particular contribute to 74-93% of these cases. Overall the number of new cases of these four cancers may be relatively high implying notable health care cost to society. The aim of this study was to estimate the incidence and the health care sector costs of anal, penile, vaginal and vulvar cancer.

**Methods:**

New anogenital cancer patients were identified from the Danish National Cancer Register using ICD-10 diagnosis codes. Resource use in the health care sector was estimated for the year prior to diagnosis, and for the first, second and third years after diagnosis. Hospital resource use was defined in terms of registered hospital contacts, using DRG (Diagnosis Related Groups) and DAGS (Danish Outpatient Groups System) charges as cost estimates for inpatient and outpatient contacts, respectively. Health care consumption by cancer patients diagnosed in 2004–2007 was compared with that by an age- and sex-matched cohort without cancer. Hospital costs attributable to four anogenital cancers were estimated using regression analysis.

**Results:**

The annual incidence of anal cancer in Denmark is 1.9 per 100,000 persons. The corresponding incidence rates for penile, vaginal and vulvar cancer are 1.7, 0.9 and 3.6 per 100,000 males/females, respectively. The total number of new cases of these four cancers in Denmark is about 270 per year. In comparison, the total number of new cases cervical cancer is around 390 per year. The total cost of anogenital cancer to the hospital sector was estimated to be 7.6 million Euros per year. Costs associated with anal and vulvar cancer constituted the majority of the costs.

**Conclusions:**

Anogenital cancer incurs considerable costs to the Danish hospital sector. It is expected that the current HPV vaccination program will markedly reduce this burden.

## Background

The introduction of two vaccines against human papillomavirus (HPV) types 16 & 18 and types 6, 11, 16 & 18, respectively, was primarily intended to protect against cervical cancer (and precancerous lesions) and genital warts. However, HPV also contributes to 40-85% of all cases of anal, penile, vaginal and vulvar cancer and precancerous lesions. HPV types 16 & 18 in particular contribute to 74-93% of these cases [1-6] and clinical studies of the quadrivalent HPV vaccine has proven protection against infection, persistent infection and low and high grade lesions in male and female populations, respectively [7-9].

Cervical cancer is the second most common cancer among women worldwide and is the fifth most frequent cancer among Danish women [10-12]. Less information is available on other HPV-related cancers but the total number of new cases of anal, penile, vaginal and vulvar cancer may be relatively high, implying considerable health care costs to society [11]. Although the incidence of cervical cancer has remained stable over the last decade [11,13], the incidence of anal and vulvar cancer has increased [11].

The aim of this study was to estimate the incidence and the health care sector cost of anal, penile, vaginal and vulvar cancer managed at the hospital level. The results will contribute to a forthcoming cost-effectiveness analysis of HPV vaccination that, besides cervical cancer and genital warts, will include the vaccine’s protection against anal, penile, vaginal and vulvar cancer.

## Methods

Patient data were extracted from Danish national registers, which are linked through individuals’ unique registration number (CPR-number). The Danish health service has a long tradition of recording health service use and each contact with primary health care (e.g. general practitioner, public and private specialist, dentist, physiotherapist) and secondary health care (e.g. hospital admission, outpatient visits) is recorded with related data on age, sex, type of contact, speciality, fee/charge, diagnoses (secondary health care only) and procedure code. The present study was approved by the Danish Data Protection Agency (J. No. 2010-41-4305).

The analysis was conducted from a hospital sector perspective, as the relevant cancer types are almost exclusively diagnosed and treated at hospitals. New cancer patients were identified via specific ICD-10 diagnosis codes in the Danish National Cancer Register. Their annual hospital resource use was estimated based on hospital contacts recorded in the National Patient Register, which defines resource use by the DRG (Diagnosis Related Groups) system for admissions and by the Danish outpatient charges (DAGS charges) for outpatient visits (including emergency unit contacts) [14]. The 2008 DRG and DAGS charges were used as cost estimates.

The cohort of cancer patients was defined as patients registered in the Danish National Cancer Register during 2004–2007 with anal, penile, vaginal or vulvar cancer as the primary localization. The patients were identified using the ICD-10 codes: C21 (anal cancer), C60 (penile cancer), C52 (vaginal cancer) and C51 (vulvar cancer).

For the cohort of cancer patients diagnosed during 2004–2007, health care use in 2006–2008 was compared with an age- and sex-matched cohort without cancer (controls). Five controls were identified for each cancer patient. The hospital costs associated with the controls were subtracted from the costs associated with the cancer patients (to identify the extra costs related to cancer), but this was done in regression analyses in which costs attributable to anal, penile, vaginal and vulvar cancer were estimated with cancer (yes/no) as an explanatory dummy variable. As a substantial number of the control patients incurred no health care costs (i.e. cost = 0), a two-part model was applied [15-17]. In this analysis, the probability that the patient had zero or non-zero costs was first predicted via logistic regression analysis. Secondly, the level of cost conditional on having positive costs was predicted using a generalized linear regression model (GLM), applying a log link function and assuming an inverse Gaussian distribution. Finally, the estimated health care costs were derived by multiplying the predictions from the two components (part 1 and 2) together.

When the data for this study was obtained, data on cancer incidence (the Danish National Cancer Register) was only available up until 2007, while data on DRG *and* DAGS charges in the National Patient Register were only available from 2006 (given that the same, and relatively novel, version of the DRG- and DAGS-charges should be applied for all years). Therefore, we used a combined cross-sectional and longitudinal approach for the data analysis, where patients diagnosed during 2004–2007 had an associated resource use for 2006–2008. This allowed us to estimate the costs 0–12 months before the date of diagnosis (e.g. 2006 resource use data for a patient diagnosed in 2007) and the costs 0–12 months, 13–24 months (e.g. 2008 resource use data for a patient diagnosed in 2006) and 25–36 months after the date of diagnosis (e.g. 2008 resource use data for a patient diagnosed in 2005). Results are thus presented as yearly cost estimates for the year before, the 1^st^ year, the 2^nd^ year and the 3^rd^ year after the date of diagnosis for patients alive at the respective times. We included costs for the year before diagnosis so that we could estimate the costs of initial examinations and diagnostics. Given the perspective of this analysis, the hospital cost estimates include medication related to hospital contacts, radiotherapy, chemotherapy and specialized rehabilitation.

Costs are presented in Euros. Future costs (i.e. cost estimates for the 2^nd^ and 3^rd^ years after diagnosis) were discounted using a 3% annual discount rate to represent their present value. When estimating the total average health care cost per patient, we adjusted for deaths during the observation period.

The number of diagnosed precancerous lesions was estimated based on data from the National Pathology Register. It was not possible to estimate treatment costs for these lesions (some of which progress to anogenital cancer) separately, however, as many of these cases are diagnosed and treated by privately practising specialists in the primary care sector. As diagnoses are not systematically registered for the primary care sector, it was impossible to distinguish contacts related to precancerous lesions from other contacts.

Data were analysed using SAS software version 9.2 (SAS Institute Inc., Cary, NC, USA).

## Results

**Figure 1:**
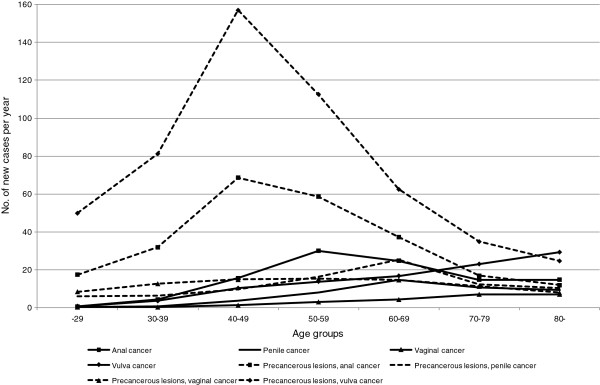
Annual incidence of anal, penile, vaginal and vulvar cancer and precancerous lesions

The incidence of the four anogenital cancers is shown in Table 1. In females, the incidence is highest for vulvar cancer and then anal cancer, and in males the incidence is highest for penile cancer and then anal cancer. For both sexes, it appears that incidence in patients under 65 years is highest for anal cancer. Figure 1 shows an increasing incidence of anal and penile cancer until age 50–59 years and 60–69 years, respectively, followed by a decrease. The incidence of vaginal and vulvar cancer increases with increasing age.

**Table 1:** Incidence of anal, penile, vaginal and vulvar cancer and precancerous lesions

	**Gender**	**Year**	**No. of cases**	**Mean age (years)**	**Medi-an age (years)**	**Min. age (years)**	**Max. age (years)**
Anal cancer (ICD10 code: C21)	Male	2004	27	61	59	33	89
	Female	2004	64	68	68	34	97
	Male	2005	25	62	64	35	85
	Female	2005	63	62	61	33	92
	Male	2006	48	59	59	18	93
	Female	2006	74	63	59	40	97
	Male	2007	42	64	62	37	91
	Female	2007	75	59	57	33	93
		Average no. cases 2004-2007	105	62			
		Incidence per 100,000 persons	1.9				
		Incidence per 100,000 males	1.3				
		Incidence per 100,000 females	2.6				
		% of patients under 65 years	60.5%				
Anal precancerous lesions, average no. of cases/year			242	52	-	-	-
Penile cancer (ICD10 code: C60)	Male	2004	39	67	66	40	93
		2005	47	68	66	38	88
		2006	46	67	66	25	94
		2007	55	68	69	40	93
		Average no. cases 2004-2007	47	67			
		Incidence per 100,000 males	1.7				
		% of patients under 65 years	44.9%				
Penile precancerous lesions, average no. of cases/year			86	60	-	-	-
Vaginal cancer (ICD10 code: C52)	Female	2004	26	74	76	46	99
		2005	15	74	75	53	92
		2006	25	76	78	49	94
		2007	26	67	73	37	95
		Average no. cases 2004-2007	23	73			
		Incidence per 100,000 females	0.9				
		% of patients under 65 years	29.7%				
Vaginal precancerous lesions, average no. of cases/year			85	55	-	-	-
Vulvar cancer (ICD10 code: C51)	Female	2004	99	70	74	30	96
		2005	93	69	70	30	96
		2006	104	68	70	23	96
		2007	93	70	71	27	103
		Average no. cases 2004-2007	97	69			
		Incidence per 100,000 females	3.6				
		% of patients under 65 years	37.5%				
Vulvar precancerous lesions, average no. of cases/year			522	50	-	-	-

Patients were followed until the end of 2008. After 24 months 74%, 75%, 47% and 71% of patients with anal, penile, vaginal and vulvar cancer, respectively, were alive. These results cannot be interpreted as 2-year survival rates, however, as patients may have died from other causes.

As could be expected, costs associated with the four cancers were highest in the first 12 months after diagnosis (Table 2). Among the many diagnostic, surgical and other treatment procedures that were registered, the most frequent were radiotherapy, gynaecological examination, X-ray examination of thorax and proctoscopy.

**Table 2:** Mean annual hospital cost (2008 prices) per patient of anal, penile, vaginal and vulvar cancer

	Year before diagnosis*, €	**1**^**st**^year**, €	**2**^**nd**^year**, €	**3**^**rd**^year**, €
	**(95% CI)**	**(95% CI)**	**(95% CI)**	**(95% CI)**
Anal cancer	4,285	23,557	7,926	5,753
	(3,260-5,612)	(18,298-30,323)	(6,119-10,265)	(4,266-7,750)
Anal cancer, men	4,524	25,335	8,567	6,498
	(2,953-6,931)	(16,248-39,420)	(5,715-12,818)	(3,921-10,736)
Anal cancer, women	4,143	22,620	7,608	5,440
	(2,991-5,735)	(16,475-31,038)	(5,585-10,364)	(3,901-7,586)
Penile cancer	2,238	12,497	3,968	3,634
	(1,758-2,644)	(9,146-17,075)	(3,034-5,149)	(2,458-5,290)
Vaginal cancer	3,789	17,154	7,215	***
	(2,394-5,986)	(9,794-29,925)	(4,189-12,425)	
Vulvar cancer	2,824	12,353	4,044	4,269
	(2,271-3,477)	(9,382-16,260)	(3,132-5,211)	(3,034-5,995)

* The average cost 0–12 months before the date of diagnosis.

** The average cost 0–12 months after the date of diagnosis, 13–24 months after the date of diagnosis for living patients, and 25–36 months after the date of diagnosis for living patients.

*** Results not estimated due to few observations.

€ 1.00 = DKK 7.45.

The estimates for the total average cost per patient, which were derived from the data in Table 2, are shown in Table 3. The total average cost per patient is thus the sum of the cost estimates for the year before and the 1^st^, 2^nd^ and 3^rd^ years after diagnosis with adjustment for death during the observation period and with future costs (2^nd^ and 3^rd^ years) discounted. Anal cancer, especially in men, incurs the highest total cost per patient. In total, the cost to the hospital sector of anogenital cancer constitutes 7.6 million Euros per year – comprising 2.4 million Euros per year for anogenital cancer in men and 5.2 million Euros per year for anogenital cancer in women. The costs associated with anal and vulvar cancer constitute the majority of the costs (53% and 27%, respectively).

**Table 3:** Total hospital costs (2008 prices) in Denmark for anal, penile, vaginal and vulvar cancer

	**Total cost per patient €**	**Total cost per patient excluding costs in the year before diagnosis €**	**Total cost per year in Denmark €**	**Total cost per year attributable to HPV 16 & 18 €**
Anal cancer	38,289	34,004	4,001,230	3,147,043
Anal cancer, men	41,347	36,822	1,467,811	1,154,461
Anal cancer, women	36,734	32,590	2,534,623	1,993,529
Penile cancer	20,513	18,275	958,972	329,610
Vaginal cancer	25,435	21,646	585,011	358,625
Vulvar cancer	21,161	18,337	2,057,936	753,254
Total costs, anogenital cancer	-	-	7,603,149	4,588,532

3% discount rate applied. € 1.00 = DKK 7.45.

Using the prevalence results presented in Table 4, we estimated costs attributable to HPV 16 and 18. The total hospital costs associated with HPV 16/18-related anogenital cancers were estimated at 4.6 million Euros per year, of which 1. 5 million Euros and 3.1 million Euros occurred in men and women, respectively (Table 3).

**Table 4:** HPV prevalence in anogenital cancer

	**HPV prevalence (% of cancers)**	**Prevalence (% of HPV-positive cancers)**		
		**HPV 16**	**HPV 18**	HPV 16/18***
Anal cancer*	84.3	87.1	6.2	93.3
Penile cancer**	46.7	60.2	13.4	73.6
Vaginal cancer*	69.9	76.8	10.9	87.7
Vulvar cancer*	40.4	79.7	10.9	90.6

* Source: [6].

** Source: [5].

*** Co-infections are not taken into account.

## Discussion

This study estimated the incidence and the hospital sector cost of anal, penile, vaginal and vulvar cancer in Denmark, using register data at individual patient level. Danish registers are comprehensive and have good recording practices, with the result that every incident anogenital cancer patient in the period 2004–2007 can be expected to be included in this analysis. The total number of new cases of these four cancers in Denmark is about 270 per year. In comparison, the total number of new cases cervical cancer is around 390 per year [11]. The total cost of anogenital cancer to the hospital sector was estimated to be 7.6 million Euros per year (2.4 million Euros per year for men and 5.2 million Euros per year for women). Costs associated with anal and vulvar cancer comprised 53% and 27% of the total cost, respectively. In comparison, the total hospital cost of cervical cancer (excluding precancerous lesions) is estimated to be 10.2 million Euros per year (2008 price level, estimated on the basis of Olsen and Jepsen, 2010 [18]). The total health care sector costs of genital warts is estimated to be 8.0 million Euros per year (2008 price level) [18].

A limited number of international publications on the cost-of-illness of anogenital cancers are available. The present cost estimates are markedly higher than those by Borget et al. (2011) [19] and Abramowitz et al. (2010) [20] but similar to US cost estimates from Hu and Goldie (2008) [21]. Discrepancies may be due to differences in cost levels between countries (especially salaries for health professionals). Other important factors, however, are differences in methodology (prevalent vs. incident patients), health service organization and clinical practice, and in time horizon for the analyses (longitudinal vs. cross-sectional approach). The strengths of this study are the use of comprehensive national registers that include all incident patients in the years 2004–2007 and the estimation of costs for four separate years. In comparison, for example, Borget et al. used a 1-year cross-sectional approach [19].

Costs were estimated using DRG and DAGS charges as unit costs for admissions and outpatient contacts, respectively. These may not accurately reflect the opportunity cost, which should ideally be used in such analyses. The charges used here were considered to be the best available proxies for the opportunity costs.

Although use of the Danish National Cancer Register and the National Patient Register is expected to have identified all incident cases of anogenital cancer, the results are likely to be an underestimation of the burden of disease and costs related to anogenital cancers. Firstly, the applied perspective ignores the productivity costs to society that are associated with anogenital cancer, such as indirect costs in terms of absence from work due to cancer-related symptoms and treatment. Although many patients had left the workforce at the time of diagnosis (the retirement age in Denmark is typically 65 years), some were likely to be still in the work force at the time of diagnosis – particularly among anal cancer patients, where 60% were aged under 65 years. This implies a considerable productivity loss to society. Secondly, the present study did not cost cases of precancerous lesions *not* progressing to cancer (from Figure 1 it can be seen that the number of cases of precancerous lesions exceeds the number of cases of genital cancer). Many precancerous lesions are diagnosed and treated by privately practising specialists in the primary care sector, and costs to primary care were not included in this study. Finally, any extra use of primary health care services by anogenital cancer patients (above that used by the general population) were not included in the analysis.

On the other hand, the costs attributable to HPV 16/18 is probably slightly overestimated as the prevalence of HPV 16 and 18 is simply added in the last column in Table 4 thereby ignoring co-infections with both these HPV types. However, co-infection with HPV 16/18 seems infrequently encountered. It has been found in 0 to 3.2% of HPV 16 positive cervical samples [22,23].

Finally, it should be noted that certain types of oropharyngeal cancers also are related to HPV 16 and 18 implying that the burden of HPV-related cancers, other than cervical cancer, is higher than reported in this study.

## Conclusions

The total cost of anogenital cancer to the hospital sector was estimated to be 7.6 million Euros per year. It is expected that the current Danish HPV vaccination program will markedly reduce this burden. This study provides the first estimate of the costs associated with non-cervical HPV-related cancers in Denmark, based on very reliable individual-level data. Future cost-effectiveness studies of the Danish HPV vaccination program should include the impact of the vaccine’s protection against anogenital cancers.

## Competing interests

J. Olsen has received speaker fees from Sanofi Pasteur MSD.

T.R. Jørgensen is employed by Sanofi Pasteur MSD.

K. Kofoed has received fees as a speaker and obtained research grants from Sanofi Pasteur MSD.

H.K. Larsen: no competing interests.

## Authors’ contributions

JO and TRJ designed the analysis. JO performed the statistical analysis and wrote the first draft. TRJ, KK and HKL contributed to the interpretation of data, drew conclusions and critically revised the manuscript. All authors read and approved the final manuscript.

## Pre-publication history

The pre-publication history for this paper can be accessed here:

http://www.biomedcentral.com/1471-2458/12/1082/prepub
